# Association between age at first calving and lactation performance, lactation curve, calving interval, calf birth weight, and dystocia in Holstein dairy cows

**DOI:** 10.1371/journal.pone.0244825

**Published:** 2021-01-04

**Authors:** Hadi Atashi, Anise Asaadi, Miel Hostens

**Affiliations:** 1 Department of Animal Science, Shiraz University, Shiraz, Iran; 2 Department of Clinical Science, School of Veterinary Medicine, Shiraz University, Shiraz, Iran; 3 Department of Farm Animal Health, University of Utrecht, Utrecht, The Netherlands; Michigan State University, UNITED STATES

## Abstract

In the present study, records on 115,291 heifers distributed in 113 herds were used to investigate the association between age at the first calving (**AFC**) and lactation performance, lactation curve, the length of the first calving interval (**CI**), calf birth weight (**CBW**), and the incidence of dystocia in Holstein heifers in Iran. Based on the AFC, the heifers were classified into eight classes: AFC of 541 to 690 d, 691 to 720 d, 721 to 750 d, 751 to 780 d, 781 to 810 d, 811 to 840 d, 841 to 900 d, and 901 to 1200 d (AFC1 to AFC8, respectively). Multiple regression mixed models were used to investigate the association between AFC and lactation curve parameters, partial and 305-d lactation performance, 100- and 305-d SCS, and the length of the first calving (CI) interval. The mean (SD) and median AFC across all heifers was 760.2 (74.01) and 750 d, respectively. Of 115,291 heifers included, 28,192 and 7,602 heifers were, respectively, ≤ 720 and > 900 d when calving for the first time. More than 44% of the heifers were at 691 to 750 d (23 to 25 months) of age when calving for the first time. An increased AFC was associated with increased partial and 305-d lactation performance, 100- and 305-d SCS, initial milk yield, milk production at the peak of lactation, upward and downward slopes of the lactation curve. The 305-d fat percentage was associated with AFC; however, there was no association between AFC and 305-d protein percentage. An increased AFC was also associated with decreased milk production persistency, delayed peak time, longer CI, and higher calf birth weight. Compared to heifers calving for the first time between 691 to 780 d (23 to 26 months) of age, both increasing and decreasing AFC were associated with increased risk of dystocia. Controlling AFC is an important management factor in achieving a lower risk of dystocia, higher lactation performance, lower SCS, and shorter length of the calving interval.

## Introduction

Age at first calving (**AFC**), the period that a female calf needs to reach puberty and to reproduce for the first time, is an important factor in the cost of rearing replacements in dairy herds [1–3]. It has been reported that the optimum AFC for Holstein dairy heifers is 23 to 24 months [3–6]. The length of rearing period has a direct effect on the total cost of heifer rearing [1, 3, 4, 6, 7]; therefore, controlling AFC can be used as a management factor to reduce rearing costs. Tozer and Heinrichs [1] reported that reducing the AFC by 30 d lowered the cost of a replacement program by 4.3%. Krpálková, Cabrera [8] considering milk yield, milk components, services per conception, days open, and the length of the calving interval (CI), recommended AFC shorter than 700 d for Holstein dairy herds with suitable management. Heifers that conceive earlier and give birth at a younger age are also more fertile in later stages and may have shorter CI length [9]. Decreasing AFC is also associated with a shorter generation interval and subsequently higher genetic progress [10]; however, calving too early may be associated with an increased risk of dystocia, reduction in milk yield, milk components and reproductive performance [5, 9, 11]. Increasing AFC, on the other hand, has been reported to be associated with a higher milk, and protein yield, as well as a lower risk of dystocia in the first lactation [10, 12, 13]. In addition, it has been showed that heifers calving at older age usually have a lower lifetime productive and reproductive performance and survive shorter than those calving at younger age [9, 12, 14–17]. Although the association between AFC and 305-d lactation performance has been well documented, the knowledge on relationship between the AFC and the shape of the lactation curve is sparse. In addition, the accumulated 305‐day lactation performance, used in most previous studies, is estimated by summing the test‐day record collected every day during the lactation period or combining the weekly or monthly test-day record by linear interpolation [18]. Furthermore, a number of researchers showed that the association between AFC and lactation performance changed during the lactation [2, 8]; therefore, the association may be quantified more accurately and in more detail using mathematical models describing the lactation curve. The aim of this study was to use an incomplete gamma function to investigate the association between AFC and lactation curve parameters, partial and 305-d lactation performance in Holstein cows in Iran. The association of AFC and 100- and 305-d somatic cell score, calf birth weight, risk of dystocia and the length of the first calving interval was also investigated.

## Materials and methods

Data used in this study were records on Holstein cows collected from January 2008 to December 2018 by the Animal Breeding Center of Iran (Karaj, Iran). First-lactation records on 128,499 dairy heifers distributed in 113 free-stall herds with sizes of 20 to 3,000 dairy cows were used to investigate the association between AFC and lactation curve parameters, partial and 305-d lactation performance. The herds evaluated were purebred Holsteins, managed under conditions similar to those used in most developed countries, and were under official performance and pedigree recording. Iranian dairy farmers monitor heifers for signs of puberty or oestrous activity such as mounting activity, evidence of hair swept backwards or missing hair on the tail head with the goal of breeding these heifers at 12 to14 months of age. The diet, fed as a total mixed ration (**TMR**), consisted of corn silage, alfalfa hay, barley grain, fat powder, beet pulp, and feed additives. Monthly milk recording was performed by trained technicians of the Iranian Animal Breeding Center, according to the guidelines of the International Committee for Animal Recording [19]. The maximum interval between the parturition and the first milk recording was 35 d and the interval between two subsequent milk recordings ranged from 22 to 37 days. Almost all cows were milked three times daily (morning, afternoon, and night). Test-day milk record was defined as the sum of production for the three milkings, beginning with the morning milking of each recording day. After the estimation of 24-hour fat and protein percentages, 24-hour fat and protein yield were computed by multiplying the milk yield and the protein and fat percent collected on the recording day. The test day samples for milk composition and somatic cell count (**SCC**) were taken simultaneously. The Fossomatic which stains cells with a fluorescent dye and then counts the number of fluorescing particles is the method used by the Animal Breeding Center of Iran to measure SCC. Averaging the somatic cell count of the morning, noon and night milk samples provides the 24-hour SCC. Test-day somatic cell count (**SCC**) were first log transformed to somatic cell score (**SCS**) based on the following equation and then averaged along 5 to 100 or 5 to 305 DIM (100-d SCS and 305-d SCS).

SCS=ln(SCC/100000)+3

Farmers, upon observing parturition, subjectively assigned a calving ease score according to the degree of assistance provided. Recognized dystocia scores were as follows: 1 = no problem, 2 = slight problem, 3 = needed assistance, 4 = needed considerable force and 5 = extreme difficulty. In this study, dystocia scores of 1 or 2 were coded as easy calving, and scores of ≥ 3 were coded as difficult calving. Heifers with missing birth date, calving date, breeding date, dry-off date, and parity number were excluded. Heifers were required to have a minimum of 5 test-day records in the period of lactation. Tests before 6 days in milk (**DIM**) or after 320 DIM were excluded. Ultimately, the data set used to describe lactation curve included 969,498 test-day records on 128,499 heifers distributed in 113 herds. The age at the first calving (**AFC**) was calculated as the difference between birth date and calving date at the first parity and was restricted to the range of 540 to 1,200 d. The length of the first calving interval was calculated as the difference between calving date at the first and second parity and was restricted to the range of 270 to 700 d [20]. In the present study an incomplete gamma function proposed by Wood [21] was used to describe the lactation curve. This function is the most popular mathematical model for describing lactation curve with a minimum number of parameters and significant relationships between its parameters with the main features of a typical lactation pattern, such as peak yield, time at peak and persistency. The function was as follows: y_t_ = at^b^e^−ct^, where *y*_*t*_ is the daily milk yield (kg/d) at DIM *t*, the variable *t* represents the length of time since calving, *e* is the Neper number, *a* is a parameter representing yield at the beginning of the lactation, and *b* and *c* are factors associated with the upward and downward slopes of the lactation curve, respectively. The incomplete gamma function was transformed logarithmically into a linear form as: ln(y_t_) = ln(a) + bln(t)–ct, and fitted to test-day milk records using a simple program written in Visual Basic (Microsoft Corp., Redmond, WA). The time at which peak lactation occurred (T_max_) was defined as: T_max_ = (b/c), expected maximum yield (y_max_) was calculated as: y_max_ = a(b/c)^b^e^−b^, lactation persistency (s) (the rate of decline in milk yield after the time at which the peak lactation occurs) was calculated as: s = −(b + 1)ln(c), and total milk yield from the time of calving up to 100, 200, and 305 DIM was calculated as: $y=a\int_{1}^{n}t^{b}e^{-ct}\;dt$, where n = 100, 200, and 305, respectively.

Based on the age at the first calving (AFC), the heifers included in this study were classified into the following eight classes [9, 22]: AFC of 541 to 690 d, 691 to 720 d, 721 to 750 d, 751 to 780 d, 781 to 810 d, 811 to 840 d, 841 to 900 d, and 901 to 1200 d (AFC1 to AFC8, respectively).

The association between calf gender and AFC was determined using a mixed linear model through the inclusion of herd, calving year, calving season, calf gender, and random effect of the dam’s sire in PROC MIXED [23]. The association between AFC and lactation curve parameters, partial and 305-d lactation performance, 100-d and 305-d SCS, and the length of the first calving interval was investigated using the following multiple regression mixed models in PROC MIXED [23].

$$y_{ijkl}=\mu +{HYS}_{i}+{AFC}_{j}+{CE}_{k}+{SI}_{l}+e_{ijkl}$$

where y is he trait being analyzed, μ is the overall mean, HYS is herd-calving year-calving season combination (**HYS**), AFC is age at first calving category, CE is calving ease (eutocia vs. dystocia) category, SI is random effect of dam’s sire and e is random residual error effect. The association between AFC and calf birth weight was determined using the explained model, but the fixed effect of calf gender, and the random effect of service sire were included into the model, and the random effect of the dam’s sire was excluded from the model as the following.

$$y_{ijklm}=\mu +{HYS}_{i}+{AFC}_{j}+{CE}_{k}+G_{l}+{SS}_{m}+e_{ijklm}$$

where y is calf birth weight, μ is the overall mean, terms μ, HYS, AFC and CE are the same for the equation above. The terms G and SS are, respectively, fixed effect of calf gender and random effect of service sire, and e is random residual effect. The model predicted adjusted means were used to compare different classes of factors including AFC, calf gender, and dystocia through LSM procedure in SAS [23]. The association between AFC and the risk of dystocia was investigated using a multivariable logistic regression model through the maximum likelihood method of PROC GENMOD [23]. In the model, the dependent variable, dystocia score, was 0 for easy calving and 1 for difficult calving, and the independent variables were herd, calving year, calving season, AFC, calf gender and random effect of service sire. Reference category for comparison of odds ratios (OR) for calf sex, calving season and AFC were, respectively, female, spring, and AFC2.

## Results

### AFC

Age at first calving distribution is shown in Table 1. The mean (SD) and median AFC across all heifers was 760.2 (74.01) and 750 d, respectively. Of 115,291 heifers included in the study, 28,192 heifers (24.5%) calved for the first time at 720 d of age or younger, while 7,602 heifers (6.6%) were older than 900 d when calving for the first time. More than 44% of the heifers were at 690 to 750 d of age when calving for the first time (AFC2 and AFC3). AFC was associated with calf gender, while predicted mean AFC was longer in heifers delivering males compared to those delivering female calves (769.1 (SE = 1.3) vs. 766.3 (SE = 1.3) d). The highest and lowest predicted mean AFC was found for heifers calving in winter and summer, respectively (771.7 (SE = 1.4) vs. 763.9 (SE = 1.4) d). The mean AFC decreased by 1.64 (SE = 0.06) d per year during 2008 and 2018.

**Table 1:** Categories, corresponding range, arithmetic means, standard deviation, and frequency distribution of age at first calving for heifers included in the study.

AFC category^1^	AFC range (d)	Mean (d)	SD (d)	Frequency (no)	Percentage (%)
AFC1	541–690	669.5	22.94	7115	6.2
AFC2	691–720	707.7	8.34	21077	18.3
AFC3	721–750	735.4	8.54	30212	26.2
AFC4	751–780	764.3	8.59	21839	18.9
AFC5	781–810	794.1	8.60	13146	11.4
AFC6	811–840	824.0	8.60	6994	6.1
AFC7	841–900	865.4	16.80	7306	6.3
AFC8	901–1200	970.3	62.61	7602	6.6

^1.^ Based on the age at the first calving (AFC), the heifers were classified into eight classes: AFC of 541 to 690 d, 691 to 720 d, 721 to 750 d, 751 to 780 d, 781 to 810 d, 811 to 840 d, 841 to 900 d, and 901 to 1200 d (AFC1 to AFC8, respectively).

### Association between AFC and partial and 305-d lactation performance

An increased AFC was associated with increased partial and 305-d lactation performance (Table 2). The lowest predicted mean 100-, 200-, 305-d milk yield, 305-d fat yield, and 305-d protein yield was recorded for AFC1 followed by AFC2 (Table 2). An increased AFC was also associated with increased 305-d milk fat percentage; however, there was no association between AFC and 305-d milk protein percentage (*P* > 0.05). Mean (SD) 100-d and 305-d SCS was 2.43 (1.03) and 2.48 (0.85), respectively. Increased AFC was also associated with increased 100-d and 305-d SCS, while the predicted means (SE) 100-d SCS were 2.38 (0.01) and 2.48 (0.01) for AFC1 and AFC8, respectively. The corresponding values for 305-d SCS were 2.44 (0.01) and 2.56 (0.02), respectively.

**Table 2:** Effects of calving ease (eutocia vs. dystocia) and first calving age on partial and 305-d lactation performance (n = 115,291).

	100-d milk (kg)^3^	200-d milk (kg)^4^	305-d milk (kg)^5^	305-d fat (kg)	305-d fat percentage (%)	305-d protein (kg)	305-d protein percentage (%)	100-SCS^6^	305-d SCS^7^
Calving ease									
Eutocia	3177(4.2)^a^	6471(9.5)^a^	9410(14.8)^a^	271.7(0.43)^a^	3.276(0.003)^b^	260.4(0.42)^a^	3.091(0.001)^a^	2.45(0.008)^a^	2.51(0.007)^a^
Dystocia	3110(6.2)^b^	6380(12.4)^b^	9308(19.3)^b^	271.9(0.65)^a^	3.287(0.005)^a^	260.3(0.63)^a^	3.093(0.003)^a^	2.42(0.011)^b^	2.49(0.011)^b^
AFC category^2^									
AFC1	3029(7.5)^f^	6225(14.6)^f^	9102(22.7)^e^	266.8(0.80)^e^	3.275(0.006)^c^	255.7(0.80)^f^	3.097(0.003)^a^	2.38(0.01)^c^	2.44(0.01)^e^
AFC2	3093(5.7)^e^	6344(11.5)^e^	9276(17.9)^d^	268.7(0.58)^e^	3.271(0.005)^c^	258.0(0.57)^e^	3.092(0.003)^a^	2.39(0.01)^c^	2.46(0.01)^d^^e^
AFC3	3130(5.4)^d^	6400(10.9)^d^	9332(19.1)^c^	270.5(0.54)^d^	3.273(0.004)^c^	259.3(0.53)^d^^e^	3.089(0.003)^a^	2.40(0.01)^c^	2.46(0.01)^c^^d^
AFC4	3140(5.6)^d^	6412(12.3)^d^	9336(17.0)^c^	271.1(0.57)^d^	3.274(0.005)^c^	259.7(0.55)^c^^d^	3.087(0.003)^a^	2.41(0.01)^b^^c^	2.47(0.01)^c^^d^
AFC5	3140(6.2)^d^	6414(11.3)^d^	9341(17.6)^c^	271.3(0.64)^c^^d^	3.276(0.005)^c^	260.6(0.62)^c^^d^	3.089(0.003)^a^	2.41(0.01)^b^^c^	2.49(0.01)^b^^c^
AFC6	3176(7.2)^c^	6481(14.1)^c^	9425(21.9)^b^	27.43(0.76)^b^^c^	3.287(0.006)^b^^c^	261.8(0.74)^b^^c^	3.096(0.004)^a^	2.43(0.01)^a^^b^	2.50(0.01)^b^
AFC7	3200(7.1)^b^	6530(14.0)^b^	9498(21.8)^a^	275.5(0.76)^a^^b^	3.293(0.006)^b^^c^	263.0(0.73)^a^^b^	3.090(0.004)^a^	2.43(0.02)^a^^b^	2.53(0.01)^a^^b^
AFC8	3240(7.7)^a^	6597(15.1)^a^	9558(23.4)^a^	277.1(0.83)^a^	3.299(0.007)^a^^b^	264.6(0.79)^a^	3.093(0.004)^a^	2.48(0.02)^a^	2.56(0.02)^a^

^a–f^ Least squares means in each column with different superscripts differ significantly (P < 0.05).

^1.^ Calculated using the following function: ln(y_t_) = ln(a) + bln(t)–ct, where *y*_*t*_ is the daily milk yield (kg/d) at DIM *t*, the variable *t* represents the length of time since calving, *e* is the Neper number, *a* is a parameter representing yield at the beginning of the lactation, and *b* and *c* are factors associated with the upward and downward slopes of the lactation curve, respectively.

^2.^ Based on the age at the first calving (AFC), the heifers were classified into eight classes: AFC of 541 to 690 d, 691 to 720 d, 721 to 750 d, 751 to 780 d, 781 to 810 d, 811 to 840 d, 841 to 900 d, and 901 to 1200 d (AFC1 to AFC8, respectively).

^3.^ Calculated as $100-d\;milk=a\int_{1}^{100}t^{b}e^{-ct}\;dt$.

^4.^ Calculated as $200-d\;milk=a\int_{1}^{200}t^{b}e^{-ct}\;dt$.

^5.^ Calculated as $305-d\;milk=a\int_{1}^{305}t^{b}e^{-ct}\;dt$.

^6.^ 100-d averaged SCS.

^7.^ 305-d averaged SCS.

### Association between AFC and the lactation curve parameters

**Fig 1:**
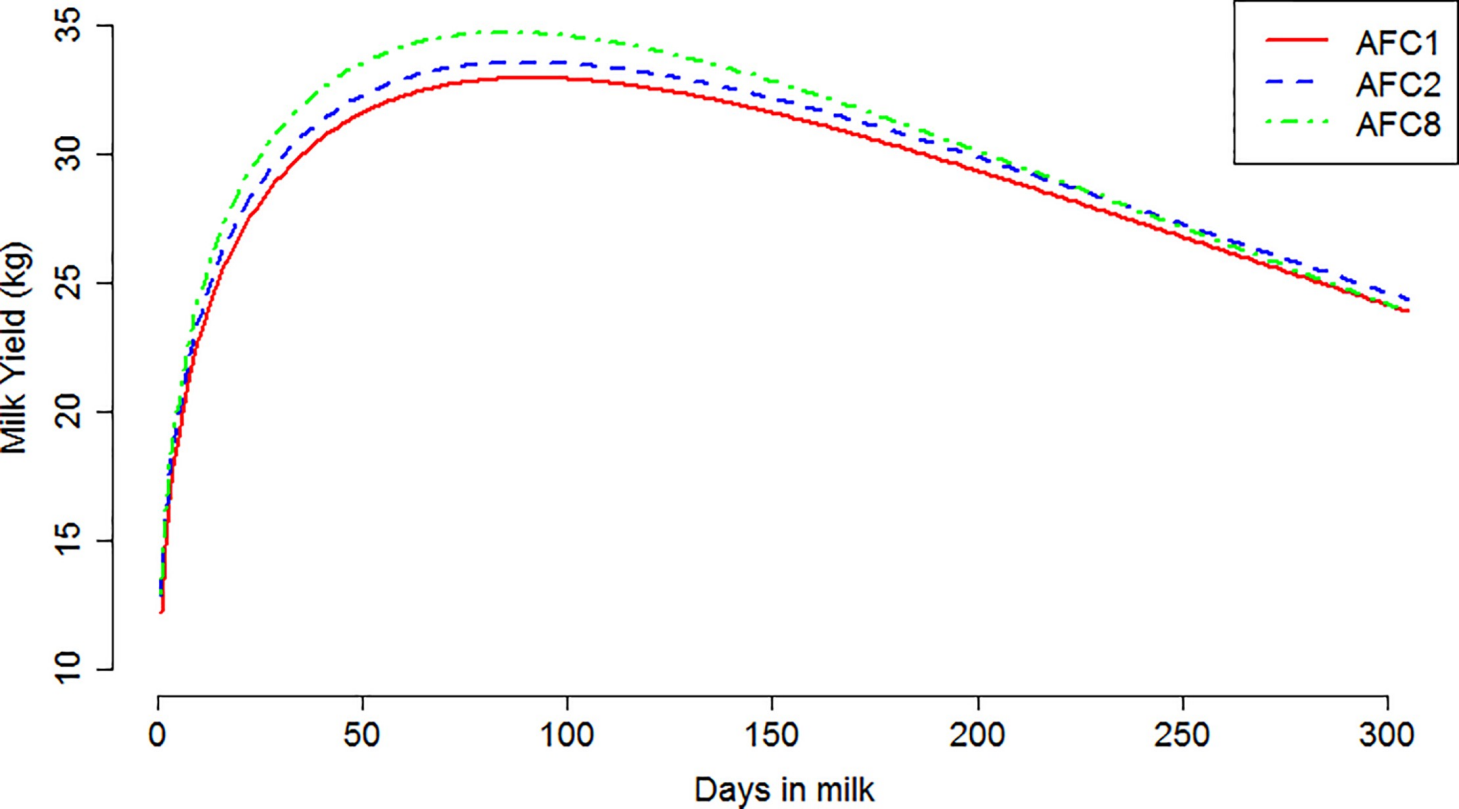
Lactation curves for heifers calving for the first time at 541 to 690 d (AFC1), 691 to 720 d (AFC2), and 901 to 1200 d (AFC8) of age.

Typical lactation curve has positive *a*, *b*, and *c*, and those curves with negatives *a*, *b*, or *c* are considered atypical. Of 128,499 heifers, 13,208 (10.3%) showed atypical lactation curve and were excluded from further analyses. The mean (SD) logarithmically transformed of the initial milk yield (ln(a)), upward slope of the lactation curve (b), and downward slope of the lactation curve (c) were 2.59 (0.651), 0.267 (0.176), and 0.00308 (0.0019), respectively. The lactation curve parameters were associated with AFC (P ≤ 0.05). The shape of the lactation curve for heifers calving at younger age tended to be lower and flatter than that for heifers calving at older age, which rise more sharply and start to decline more rapidly (Fig 1). The lowest initial milk yield was recorded for heifers belonged to AFC1 followed by AFC2; however, there was no significant difference for initial milk yield among the rest AFC groups (Table 3). Although the upward slope of the lactation curve was associated with the AFC, the pattern of the association was not linear, while the lowest upward slope was found for AFC3 (Table 3). An increased AFC was also associated with increased downward slope of the lactation curve, increased amount of milk yield at the peak, decreased milk production persistency as well as delayed peak time (Table 3).

**Table 3:** Effects of calving ease (eutocia vs. dystocia) and first calving age on the lactation curve parameters, calf birth weight, and length of the first calving interval (CI) in Holstein heifers (n = 115,291).

	Ln(a)	b	c	s^5^	Peak yield (kg)^6^	Peak day (d)^7^	Calf birth weight (kg)^6^	CI(d)
Calving ease								
Eutocia	2.602(0.004)^a^	0.269(0.001)^b^	0.00313(0.00001)^b^	7.47(0.004)^b^	35.31(0.05)^a^	88.6(0.246)^b^	------------	406(0.61)^b^
Dystocia	2.528(0.007)^b^	0.283(0.002)^a^	0.00317(0.00002)^a^	7.52(0.006)^a^	34.88(0.07)^b^	92.2(0.394)^a^	------------	416(1.03)^a^
AFC category^4^								
AFC1	2.509(0.009)^c^	0.281(0.002)^a^^b^	0.00309(0.00002)^c^^d^	7.55(0.010)^a^	33.99(0.08)^g^	93.9(0.51)^a^	39.8(0.07)^e^	407(1.29)^c^
AFC2	2.559(0.006)^b^	0.273(0.001)^c^	0.00304(0.00002)^d^	7.52(0.008)^b^	34.57(0.07)^f^	92.5(0.47)^a^	40.0(0.05)^d^	409(0.87)^c^
AFC3	2.584(0.006)^a^	0.269(0.001)^c^^d^	0.00305(0.00002)^d^	7.49(0.008)^c^	34.86(0.06)^e^	90.6(0.47)^b^	40.3(0.04)^c^	409(0.90)^c^
AFC4	2.579(0.006)^a^^b^	0.272(0.001)^c^	0.00312(0.00002)^b^^c^	7.49(0.008)^c^	34.99(0.06)^d^	89.9(0.39)^b^^c^	40.3(0.04)^c^	408(0.81)^c^
AFC5	2.569(0.007)^a^^b^	0.275(0.001)^b^^c^	0.00316(0.00002)^b^^c^	7.49(0.009)^c^	35.02(0.07)^d^	89.6(0.34)^b^^c^	40.6(0.07)^b^	411(1.00)^b^^c^
AFC6	2.573(0.009)^a^^b^	0.277(0.002)^b^^c^	0.00321(0.00003)^b^	7.48(0.010)^c^	35.42(0.07)^c^	89.2(0.32)^c^^d^	40.6(0.05)^b^	413(1.22)^b^
AFC7	2.585(0.009)^a^	0.276(0.002)^b^^c^	0.00319(0.00003)^b^	7.48(0.010)^c^	35.68(0.07)^b^	89.4(0.35)^c^^d^	40.7(0.07)^b^	414(1.22)^b^
AFC8	2.566(0.009)^a^^b^	0.285(0.002)^a^	0.00335(0.00003)^a^	7.47(0.012)^c^	36.24(0.08)^b^	88.0(0.49)^d^	41.0(0.08)^a^	420(2.36)^a^

^a–g^ Least squares means in each column with different superscripts differ significantly (P < 0.05).

^1.^ Modeled as: ln(y_t_) = ln(a) + bln(t)–ct, where *y*_*t*_ is the daily milk yield (kg/d) at DIM *t*, the variable *t* represents the length of time since calving, *e* is the Neper number, *a* is a parameter representing yield at the beginning of the lactation, and *b* and *c* are factors associated with the upward and downward slopes of the lactation curve, respectively.

^2.^ Based on the age at the first calving (AFC), the heifers were classified into eight classes: AFC of 541 to 690 d, 691 to 720 d, 721 to 750 d, 751 to 780 d, 781 to 810 d, 811 to 840 d, 841 to 900 d, and 901 to 1200 d (AFC1 to AFC8, respectively).

^3.^ lactation persistency (s) was calculated as: s = −(b + 1)ln(c).

^4.^ Expected maximum yield was calculated as: a(b/c)^b^e^−b^.

^5.^ The time at which peak lactation occurred was defined as (b/c).

^6.^ Calf birth weight of the heifers included in the study.

### Association between AFC and the length of the first calving interval, calf birth weight, and dystocia

The association between AFC and the length of the first calving interval (CI) is presented in Table 3. The mean (SD) and median CI was 404 (82.5), and 379, respectively. The result showed that an increased AFC was associated with increased length of the CI, while the predicted means (SE) CI were 407 (SE = 1.3) and 420 (SE = 2.4) for AFC1 and AFC8, respectively (*P* ≤ 0.05). The length of the CI was also associated with dystocia, while the predicted means (SE) CI were 406 (0.6) and 416 (1.0) d, respectively, for heifers belonged to eutocia and dystocia groups.

The mean (SD) calf birth weight (CBW) was 40.2 (4.86) kg. The percentage of male and female was 46.2 and 53.8%, respectively. The predicted means (SE) birth weight for male and female calves were 41.6 (0.04), and 39.2 (0.04) kg, respectively (*P* ≤ 0.05). Increased AFC was associated with increased CBW, while the predicted means (SE) CBW were 39.8 (0.07) and 41.0 (0.08) for AFC1 and AFC8, respectively (Table 3).

Odds ratios and corresponding confidence intervals for the association of AFC with risk of dystocia are shown in Table 4. The incidence of dystocia was 10.6%, which was associated with herd, calving year, calf gender, and dam’s age at the first calving (*P* ≤ 0.05). The incidence of dystocia for calving season of spring, summer, autumn and winter were, respectively, 10.5, 10.21, 10.35, and 11.4% (*P* > 0.05). Compared to heifers calving for the first time between 691 to 780 d of age (23 to 26 months), both increasing and decreasing AFC were associated with increased incidence of dystocia (Table 4). The risk of dystocia was more in heifers delivering male than that in those delivering female calves (13.7 vs. 7.9%; odds ratio ± (0.95 CI) = 1.95 (1.82–2.09) for heifers delivering male calves than that in those delivering female calves).

**Table 4:** Odds ratios (OR) and 95% confidence interval (CI) for the effects of calf sex, and age at the first calving on the incidence of dystocia in Holstein cows (n = 112,443).

Variable	No. of births	Dystocia (%)	Odds ratio (95% CI)	P-value
Calf sex				*P* ≤ 0.05
Male	52,957	13.7	1.95 (1.82–2.09)	
Female	59,486	7.9	OR reference value	
Age at first calving^1^				*P* ≤ 0.05
AFC1	5,934	11.6	1.16(1.01–1.33)	
AFC2	19,280	8.9	OR reference value	
AFC3	29,956	9.7	0.99(0.90–1.09)	
AFC4	23,041	10.9	1.03(0.93–1.14)	
AFC5	13,777	12.1	1.16(1.05–1.29)	
AFC6	7,504	12.2	1.15(1.02–1.30)	
AFC7	7,595	11.9	1.15(1.01–1.38)	
AFC8	5,356	11.5	1.26(1.06–1.44)	

^1.^ Based on the age at the first calving (AFC), the heifers were classified into eight classes: AFC of 541 to 690 d, 691 to 720 d, 721 to 750 d, 751 to 780 d, 781 to 810 d, 811 to 840 d, 841 to 900 d, and 901 to 1200 d (AFC1 to AFC8, respectively).

## Discussion

Typical lactation curve has positive *a*, *b*, and *c*, characterized by a first ascending phase from parturition till the lactation peak, followed by a second declining slope that ends with the drying-off. An atypical lactation curve is a curve with negative estimates of a, b, or c. Negative estimates of *a* implies that the initial milk yield is less than zero. A negative estimate of b with a positive estimate of c defines a downhill-shaped lactation curve. Negative b and c define a lactation curve with an initial decreasing phase to a minimum followed by an increase [24–27]. In this study, of 128,499 heifers, 13,208 (10.3%) showed atypical lactation curve. Rekik and Gara [28] reported 15% to 42% atypical curves in dairy herds of Tunisia. Atashi, Zamiri [29] and Tekerli, Akinci [30] reported 23.1% (of 85,816) and 26.3% (of 1,278) atypical curves in Holstein dairy cows in Iran and Turkey, respectively. Macciotta, Dimauro [25] considered the time from parturition to the first recorded test, as the most important factor affecting the incidence of atypical lactation curves.

This study showed that more than 44% of Holstein dairy heifers calved at 690 to 750 d of age (23 to 25 months) and only 6.6% were 900 d or older at the first calving. The average AFC in this study (760.2 d) is less than that reported in a smaller scale Iranian Holstein cows in which the average AFC was 805 d [12]. Atashi, Zamiri [31] reported that mean AFC in Holstein cows in Iran was 801.5 and that AFC decreased by 3.15 d per year, from 1994 to 2008. Eastham, Coates [9] reported that only 20.1% of the UK Holstein-Friesian dairy heifers calved at 690 to 750 d of age and that 40.9% were 900 d or older at the first calving. Pirlo, Miglior [10] reported that the mean AFC for Italian Holsteins is 866 d, while Ettema and Santos [2] reported that the most heifers had AFC of 650 to 800 d in Californian dairy herds. Froidmont, Mayeres [32] reported that only 24% of Holstein dairy cows in Wallonia, Belgium, had their first calving before 26 months of age. Hutchison, VanRaden [22] reported that mean AFC in the US Holstein is 735 d. Wathes, Brickell [11] reported that poor growth rates, poor health and inappropriate nutrition during the rearing period often lead to increased AFC. Krpálková, Cabrera [8] reported that the overall mean AFC was 728 d and that pre- and postpubertal growth had significant effect on AFC, while a higher body weight at 14 months of age led to a lower AFC. Although, it has been reported that the heritability of AFC is enough for genetic selection [12, 33, 34], there is a confounding between the genetic merit of the animal and the actual management decision to delay first insemination of the heifer. Heifers calving at 690 d or younger were predicted to produce less partial and 305-d milk than those calving at older ages which is in line with other studies [2, 8–10, 12]. The negative effect of early calving on milk yield may be attributed to low body weight in younger heifers at the start of the lactation [10]; however, no information was available on a population basis to consider the effect of body weight in this study. Assuming that animals that calve at a younger age are indeed smaller, the lower milk production observed in first lactation is likely at least in part due to diversion of more nutrients towards growth vs. production relative to older, and presumably bigger heifers. Although increased AFC was associated with increased lactation performance, extending AFC beyond 840 d did not increase partial or 305-d lactation performance. Heinrichs and Vazquez-Anon [35] reported that heifers calving at ≥ 780 d of age produced similar amounts of 305-d milk as did heifers calving at 720 d of age. Ettema and Santos [2] also reported that increasing the average AFC from 724 to 791 d resulted in no advantages in milk yield in Holstein heifers. Nilforooshan and Edriss [12] reported that increasing AFC to 24 months increased 305-d milk yield but increasing AFC beyond 24 months decreased 305-d milk yield.

The association between AFC with 100- and 200-d milk yield was stronger than that for 305-d milk yield. Krpálková, Cabrera [8] reported that increased AFC was associated with increased milk yield but only in the first 100 d of the lactation. In addition, an increased AFC was associated with increased initial milk yield, milk production at the peak, upward and downward slopes of the lactation curve, decreased milk production persistency, and delayed peak time. Ettema and Santos [2] reported that milk production was not affected by AFC during early lactation, however, compared to heifers calving at an older age, heifers calving at younger age produced less milk after 50 DIM. Increased AFC was associated with increased 305-d fat and protein yield which is in line with previous studies [2, 36]. The results showed that an increased AFC is associated with an increased milk fat percentage, however there was no association between AFC and milk protein percentage. Higher AFC has been reported to be associated with a higher milk fat percentage [2, 10, 12]. Ettema and Santos [2] reported that concentrations of fat in milk was associated with AFC in early lactation, while heifers with higher AFC had higher milk fat percentage. Krpálková, Cabrera [8] reported that increased AFC was associated with increased 100-d milk fat percentage in the first parity. The association between AFC and milk protein percentage has been investigated in a number of studies; however, the results are inconsistent [2, 10]. Higher AFC has been reported to be associated with a lower milk protein percentage [8, 10]. Krpálková, Cabrera [8] reported that increased AFC was associated with decreased 100-d milk protein percentage in the first parity but increased lifetime average milk protein percentage. Ettema and Santos [2] reported that milk protein percentage in heifers with low AFC was higher than that in heifers with medium AFC but did not differ from that in heifers with high AFC. Although, more research is needed to understand the different relationships of AFC with milk fat and protein percentages, it can be attributed to the different abilities of young and old heifers to ingest forages or concentrates resulting in different impacts on milk composition [10]. The average 100-d and 305-d SCS in this study was in line with those reported previously in Iranian Holstein cows [37]. Significant association was found between increased AFC and increased 100- and 305-d SCS. Eastham, Coates [9] also reported significant association between AFC and 305-d SCC. The association between AFC and SCS changed during the lactation, while the association between AFC and 305-d SCS was stronger than that between AFC and 100-d SCS. Heifers with good health during the rearing period often have lower AFC which could then be associated with more resilience to mastitis which can explain the association between AFC and SCC. However, Ettema and Santos [2] reported that incidence of mastitis was lower for heifers calving at 701 to 750 d, compared to those calving at younger than 700 or older than 751 d of age. Our results showed that increased AFC was associated with increased length of the calving interval which is in line with previous studies [9, 32, 38]. Krpálková, Cabrera [8] reported that increased AFC was associated with increased days open but the length of the first calving interval was not associated with AFC. Heifers with good pre- and postpubertal growth, good health and appropriate nutrition during the rearing period often have lower AFC which could then be associated with subsequent higher fertility and a lower calving interval [9].

Calf birth weight was associated with calf gender, while male calves were heavier than female calves in line with previous studies [29, 39]. The increased AFC was associated with an increased calf birth weight. Kamal, Van Eetvelde [39] also reported that the calf birth weight was heavier when the calves were born to young or standard ages heifers in comparison to calves born to old heifers.

The incidence of dystocia was 10.6% which was affected by factors including herd, calving year, calving season, calf gender, and dam’s age at the first calving in line with previous studies [40–43]. Compared to heifers calving for the first time between 691 to 780 d (23 to 26 months) of age, both increasing and decreasing AFC were associated with increased risk of dystocia. Although, it is well documented that calving too early is associated with an increased risk of dystocia [5, 13], very few studies reported an association between increased AFC and increased risk of dystocia [44]. Mee [44] reported calf birth weight and maternal pelvic size as two important risk factors accounting for 50 and 5–10% of the phenotypic variance in dystocia, respectively. Therefore, the association between increased AFC and increased risk of dystocia can be partly attributed to increased calf birth weight when extending AFC beyond 26 months of age [41, 42, 44, 45]. The association between increased risk of dystocia and decreased AFC before 23 months can be, at least in part, attributed to maternal pelvic size. However, Ettema and Santos [2] reported that calving difficulty was not associated with AFC.

## Conclusion

The higher incidence of dystocia in primiparous animals calving outside the 691 to 780 d age window (23 to 26 months) indicates that there is an intermediate optimum AFC with regards to risk of dystocia. lower age at first calving was shown here to be associated with shorter length of the calving interval; however, decreasing AFC before 27 months showed no significant association with the lenghth of the subsequent calving interval. First lactation milk production was lower among younger heifers but lactation persistency was significantly increased. Controlling AFC is an important management factor in achieving a lower risk of dystocia, higher lactation performance, lower SCS, and shorter length of the calving interval. Heifers were required to have a minimum of 5 test-day records in the period of lactation to be included in evaluating the association between AFC and lactation performance, lactation curve, calving interval, calf birth weight, and dystocia. This restriction omitted those animals that didn’t survive long enough to experience 5 test-day records form the associations. Therefore, the results should be interpreted with caution.

## Supporting information

S1 Dataset Records on 115,291 heifers distributed in 113 herds were used to investigate the association between age at the first calving (AFC) and subsequent performance in Holstein heifers in Iran. (XLSX)
